# scAEGAN: Unification of single-cell genomics data by adversarial learning of latent space correspondences

**DOI:** 10.1371/journal.pone.0281315

**Published:** 2023-02-03

**Authors:** Sumeer Ahmad Khan, Robert Lehmann, Xabier Martinez-de-Morentin, Alberto Maillo, Vincenzo Lagani, Narsis A. Kiani, David Gomez-Cabrero, Jesper Tegner

**Affiliations:** 1 Biological and Environmental Science and Engineering Division, King Abdullah University of Science and Technology (KAUST), Thuwal, Saudi Arabia; 2 Translational Bioinformatics Unit, Navarrabiomed, Complejo Hospitalario de Navarra (CHN), Universidad Pública de Navarra (UPNA), IdiSNA, Pamplona, Spain; 3 Department of Oncology and Pathology, Algorithmic Dynamic Lab, Karolinska Institute, Stockholm, Sweden; 4 Department of Medicine, Unit of Computational Medicine, Center for Molecular Medicine, Karolinska Institutet, Karolinska University Hospital, Stockholm, Sweden; 5 Mucosal and Salivary Biology Division, King’s College London Dental Institute, London, United Kingdom; 6 Computer, Electrical and Mathematical Sciences and Engineering Division, King Abdullah University of Science and Technology (KAUST), Thuwal, Saudi Arabia; 7 Science for Life Laboratory, Solna, Sweden; Georgia Institute of Technology, UNITED STATES

## Abstract

Recent progress in Single-Cell Genomics has produced different library protocols and techniques for molecular profiling. We formulate a unifying, data-driven, integrative, and predictive methodology for different libraries, samples, and paired-unpaired data modalities. Our design of scAEGAN includes an autoencoder (AE) network integrated with adversarial learning by a cycleGAN (cGAN) network. The AE learns a low-dimensional embedding of each condition, whereas the cGAN learns a non-linear mapping between the AE representations. We evaluate scAEGAN using simulated data and real scRNA-seq datasets, different library preparations (Fluidigm C1, CelSeq, CelSeq2, SmartSeq), and several data modalities as paired scRNA-seq and scATAC-seq. The scAEGAN outperforms Seurat3 in library integration, is more robust against data sparsity, and beats Seurat 4 in integrating paired data from the same cell. Furthermore, in predicting one data modality from another, scAEGAN outperforms Babel. We conclude that scAEGAN surpasses current state-of-the-art methods and unifies integration and prediction challenges.

## Introduction

The maturation of the single-cell genomics field has produced methods to profile multiple data modalities, such as single-cell RNA sequencing (scRNA-seq) and chromatin profiles (scATAC-seq), even on the same cells at the same time. This development has provided rich opportunities for a deep understanding cell states and transitions while presenting severe computational challenges [1]. One of the most notable challenges is the integration of different single-cell datasets. Integrating different experiments has proved daunting even when using the same library protocol and omics type. For example, distinct scRNA-seq datasets may differ in the number of sampled cells and sequencing depth allocated to each cell, even by several orders of magnitude. The next challenge is combining scRNA-seq data from different library protocols or species [2]. A third challenging task is integrating other data modalities from the same experiment but originating from separate cells, a case known as unpaired multi-omics integration. Finally, recent technological advances produce paired multi-omics data collected from the same cell. These challenges have thus far been addressed one by one. For example, Seurat3 [3] and MOFA+ [4] integrate unpaired data, whereas Seurat4 [5], and MultIVI [6] integrate paired data, and Babel [7] predicts one modality from another. Methods such as scAlign [8], Harmony [9], and Seurat3 target scRNA-seq datasets originating from different experiments that used the same platform [10].

In contrast, Liger [11], iMAP [12], scMerge [13], and Seurat3 can integrate datasets produced using different library protocols. Most of these limitations of only being able to target a single challenge directly derive from the internal operation of each method. Seurat3 is based on the concept of “anchors”, which are cross-dataset pairs of cells with similar biological states. This approach does not readily scale to large datasets and performs poorly when integrating heterogeneous datasets [14]. Worse, only a fraction of cell types are usually shared across datasets, making identification increasingly challenging using anchors [15]. Babel, a machine learning method, targets only gene prediction for paired data. Thus, by design, it lacks clustering capabilities and cannot tackle unpaired data or different library protocols.

Furthermore, these approaches implicitly assume that differences between datasets arise entirely from technical variation, thus potentially masking the biological signal. For example, the Mutual Nearest Neighbors (MNNs) method [16] effectively reduces differences between datasets. An alternative strategy is exemplified by Seurat3, which forces all datasets into a shared latent space. However, both dataset similarities and differences in many kinds of analysis are biologically meaningful. Thus, it requires respecting each sample’s uniqueness, protocol, and data type.

There is a need for scalable and robust integrative methods for omics data. Preferentially general enough to encompass multiple integration tasks in one systematic framework. From this standpoint, we can also expect the scale and the number of different data modalities to increase further [17].

Here we present a novel integrative method that has been designed to take these requirements into account. The critical insight motivating our approach is that we do not force all experimental samples into a single joint representation, regardless of their library protocol, data modality, paired or unpaired design. Instead, we use an autoencoder (AE) to represent and respect the distributional characteristics of each dataset and condition. The integration is performed in the latent space by learning a mapping between the different latent space representations. Inspired by recent progress in image-to-image translation, we use a cycleGAN (cGAN) architecture for obtaining a translation between the latent spaces corresponding to different datasets. Conceptually, our method reformulates the integration challenge from a problem to be addressed in raw data space into a learning challenge between different data-specific latent space representations. We denote our method scAEGAN, a coupled AE—cycleGAN architecture. Our results demonstrate that scAEGAN can target single-cell multi-omics integration tasks with performances similar to or superior to other state-of-the-art tools. Furthermore, we provide evidence that the mapping between different latent spaces is essential for effective integration by contrasting scAEGAN against the simplified approach of directly concatenating latent spaces, which forces the data into a shared latent space without learning a mapping.

## Material and methods

### Neural network architecture

scAEGAN is a unifying architecture combining AE [18] and cGAN [19]. AE, an unsupervised deep neural network, learns essential latent features and ignores the non-essential sources of variations, such as random noise [20]. Hence, the high dimensional ambient space is compressed, capturing the underlying proper data manifold.

First, each given dataset is provided as input to an AE in a matrix *X*, where rows (m) represent the cells and columns (n) indicate genes/transcripts. The AE task involves learning the encoding representation through an encoding function *e*(*x*) and then mapping back *e*(*x*) to the original input space through a decoding function *d*. For faster convergence and better accuracy, Rectified Linear Unit (ReLU) has been used as an activation function, which is given as a function *f* applied to the input *x*:

$$f\left(x\right)=max\left(x,0\right)$$

The first hidden layer *Hidden*_1_ with *l*_1_ nodes following the input *X*_i_ (row vector) is formulated as follows:

$$Hidden_{1}=f\left(w_{1}X_{i}^{T}+b_{1}\right)$$

The weight matrix *w*_1_ is of *l*_1_ × *n* dimensions and the bias term *b*_1_ is *l*_1_ length vector. Each subsequent middle layer *k* is formulated as:

$$Hidden_{k}=f\left(w_{k}Hidden_{k-1}+b_{k}\right)$$

The composition of *e* and *d*, i.e., *d*(*e*(*x*)) = *X*′ is called the reconstruction function, and the reconstruction loss function penalizes the error made, which is given as:

$$L\left(X,X^{\prime }\right)=∥X-X^{\prime }{∥}^{2}$$

The low-dimensional space representation from the AEs captures the underlying manifold of the data. Secondly, we utilize a cGAN to learn relationships between the different domains/datasets (A and B). Specifically, learning two generative mapping functions *G*_*AB*_: *A*-> *B* and *G*_*BA*_: *B*-> *A*. In addition to these generative functions, two discriminators *D*_*A*_ and *D*_*B*_ were used to regularise the generators to generate samples from a distribution close to the latent representation of A or B. We used the Wasserstein GAN adversarial loss introduced in [21]. In the Wasserstein GAN, the discriminator is replaced by a critic model. The function of the critic is not directly to separate fake samples apart from the real ones. Instead, it is trained to learn a *K*-Lipschitz continuous function, making the neural network gradient smaller than a threshold value *K*, such that ‖∇*f*‖ ≤ *K*. The primary rationale for applying this condition is that gradient behaves better, making generator optimization easier [22]. As the loss function decreases in training, the Wasserstein distance gets smaller, and the generator model’s output grows closer to the actual data distribution. This loss ensures that the generator generates the samples from a distribution close to the distribution of *B* denoted by $b∼p_{data}^{\left(b\right)}$. This Wasserstein GAN adversarial loss is applied to both the mapping functions and the objective is expressed for *G*_*AB*_: *A*-> *B*:

$$L_{GAN}\left(G_{AB},D_{B},A,B\right)=E_{b∼p_{data}^{\left(b\right)}}\left[f_{w}\left(b\right)\right]-E_{a∼p_{data}^{\left(a\right)}}\left[f_{w}\left(G_{AB}\left(a\right)\right)\right]$$

where function *f* is a K-Lipschitz continuous function, {*f*_*w*_}_*w*∈*W*_, parameterized by *w*, $a∼p_{data}^{\left(a\right)}$ represents the probability distribution of domain *A* and $b∼p_{data}^{\left(b\right)}$ denotes the probability distribution of domain *B*. The cycle consistency loss ensures that the learned mappings are cycle consistent, i.e., bringing back to the original domain. It acts as a regularization and reduces the space of possible mapping functions. Which is given as:

$$L_{cyc}\left(G_{AB},G_{BA}\right)=E_{a∼p_{data}^{\left(a\right)}}[\left|\left|G_{BA}\left(G_{AB}\left(a\right)\right)-a\right|{|}_{1}+E_{b∼p_{data}^{\left(b\right)}}[\right|\left|G_{AB}\left(G_{BA}\left(b\right)\right)-b\right|{|}_{1}$$

To train the cGAN on the latent subspaces of the two domains, the entire objective function is:

$$L\left(G_{AB},G_{BA},D_{A},D_{B}\right)=L_{GAN}\left(G_{AB},D_{B},A,B\right)+L_{GAN}\left(G_{BA},D_{A},B,A\right)\;+\lambda_{1}L_{cyc}\left(G_{AB},G_{BA}\right)+\lambda_{2}L_{ident}\left(G_{AB},G_{BA}\right)$$

$$G_{AB}^{*},G_{BA}^{*}=argmin_{G_{AB},G_{BA}}max_{D_{A},D_{B}}\;L\left(G_{AB},G_{BA},D_{A},D_{B}\right)$$

**Fig 1:**
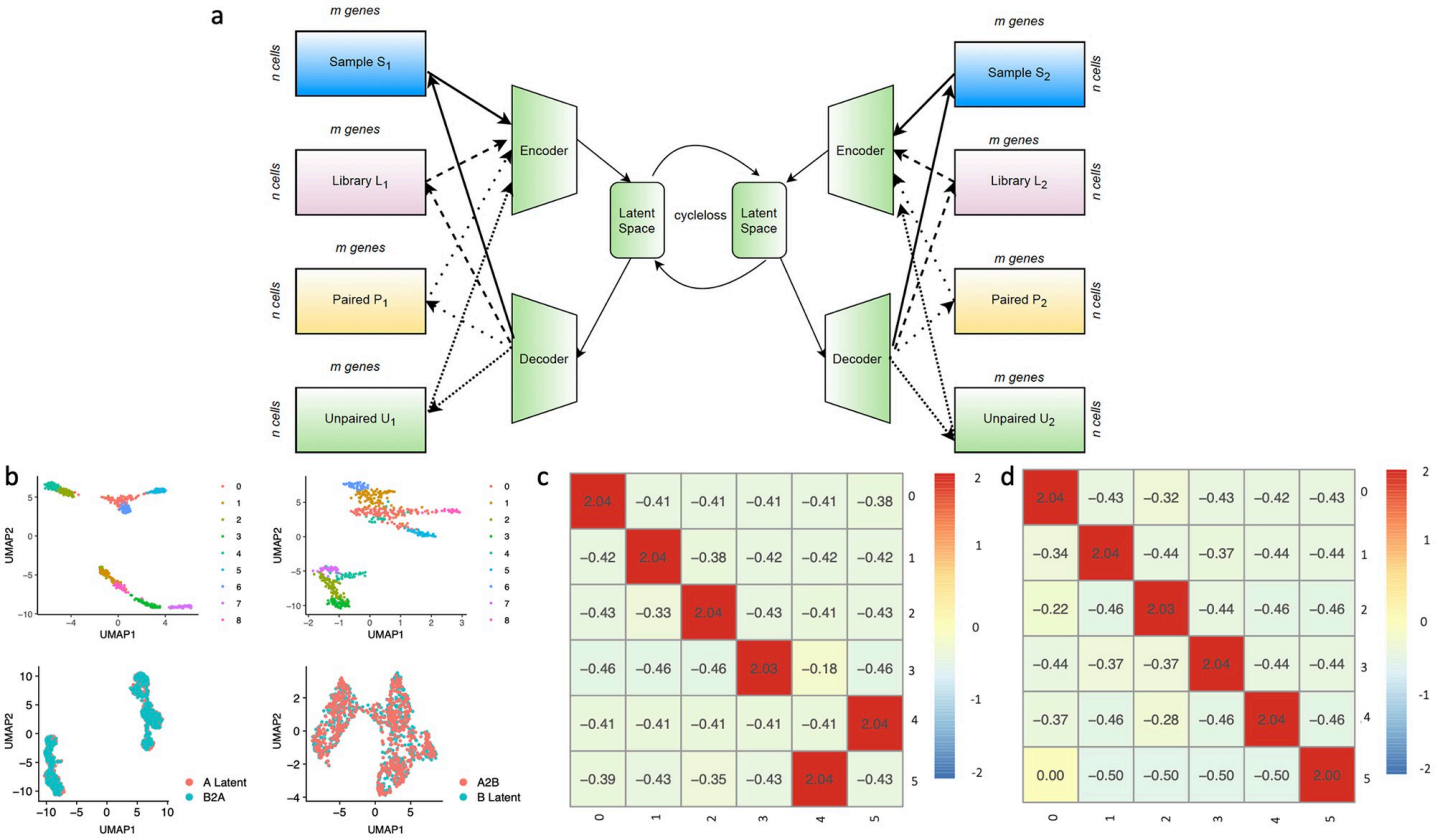
scAEGAN architecture for single cell data integration **a)** Coupled scAEGAN, allowing the translation of AE-obtained low-dimensional embeddings via a cGAN, **b)** Outputs from the scAEGAN, where A2B and B2A are integration results of the A and B datasets mapped with latent space of input datasets A and B (lower panel), **c, d)** shows the heatmaps of the cluster similarity, where x axis represents the input domain clusters and y axis represents the translated domain clusters, scAEGAN preserved the transferred cell identity agreement with the original identity.

The scAEGAN architecture is provided with a scRNA-seq and a scATAC-seq data set (domain A and B, respectively) as illustrated in Fig 1a. Each block on the left side and right side in Fig 1a represents data from domain A and domain B, respectively (for instance, Sample S_1_ represents dataset1 from the same modality and same library protocol. Sample S_2_ represents dataset2 from the same modality same protocol; likewise, for Library L_1_, L_2_ represents data from the same modality but different library protocols. The different types of lines in Fig 1a (bold and dashed) represent the input to the encoders and output from the decoder from the respective domains A and B. For instance, a bold line from Sample S_1_ represents the input to the encoder, the same bold line from the decoder to Sample S_1_ represents the reconstructed output, and likewise for other domains with a different representation of dashed lines. The first step in the scAEGAN integration algorithm is training an AE independently on both domains A and B to find a low-dimensional embedding that preserves each domain’s key defining features. This step is necessary since direct translation between scRNA-seq domains via cGAN, while possible, is hampered by increased technical variation or dataset complexity. AE is particularly suitable due to its ability to reduce random noise while still maintaining essential features. Moreover, it turns out that AE generates more biologically meaningful embeddings compared to variational autoencoders (VAE) when learning across latent spaces, which is most likely due to a poor match between the unimodal prior and the inherently multimodal scRNA-seq data [23]. A cGAN is then trained on the low-dimensional representations to achieve the translation between domains.

### Hyperparameter tuning

We have performed a series of analyses to generate the best configuration for scAEGAN hyperparameters based on the nature of the single-cell data, that resulted in the optimal configuration. The hyperparameters to be adjusted for scAEGAN are batch size, learning rate, the embedding space dimensions, and set of weighted parameters used to control the cGAN loss, i.e *λ*_1_, *λ*_2_ and training epochs.

#### AE hyperparameter and optimization

The AE model consists of three hidden layers with the dimensions of *Hidden*_1_ (300), *Hidden*_2_ (50), *Hidden*_3_ (300). ReLU has been used as an activation function for the hidden layers followed by a linear activation function in the bottleneck layer. The embeddings from this bottleneck layer are used as input to the cGAN. A dropout value of 0.2 has been used to prevent overfitting. We used Adam [24] as an optimizer with different settings ranging from lr = 0.0001 to 0.0005 and found 0.0001 as the best setting for our experiments to train the AE model. We trained the AE using a batch size of 16 for the number of epochs ranging from 60 to 200 and observed that the model trained for 120 epochs gives better performance, with 80% training and 20% validation data to analyze the convergence of the model.

#### cGAN hyperparameter and optimization

The architecture of cGAN consists of two generators and two discriminators. The generators consist of one residual block and one dense layer of 50 dimensions each, and the discriminators consist of two dense layers. A dropout value of (0.2) has been used for the residual block, followed by batch normalization, which stabilizes the learning process. In addition to this, batch normalization has a slight regularization effect; for this reason, we have used a small value (0.2) for the dropout. LeakyReLU [25] has been used as an activation function. To train the cGAN model, we have used two different optimized settings of Adam optimizer for the real data and simulated data. For the actual data, the cGAN is trained with Adam optimizer with parameters: *lr* = 0.0005 *and r* = 0.0002, *beta*_1_ = 0.5, *beta*_2_ = 0.999, *epsilon* = 1*e* − 7, *decay* = 0. And for the simulated data, the cGAN is trained with Adam optimizer with parameters: *lr* = 0.0002, *beta*_1_ = 0.5, *beta*_2_ = 0.999, *epsilon* = 1*e* − 6, *decay* = 0.0. We use the hyperparameters as weights for the cyclic loss and identity loss in all our experiments, i.e., *λ*_1_ = 0.3 and *λ*_2_ = 0.3, which were chosen to check a couple of combinations for verifying that our optimization process generates the translated data similar to the starting ones. Also, to maintain the K-Lipschitz continuity of *f*_*w*_ we used the hyperparameter *c* = 0.1 during the training, which helps in resulting in compact parameter space. In addition to these, the cGAN is trained with a batch size of 4 for 200 to 400 epochs.

### AE concatenated (AE-Concat)

The AE concatenated architecture is used for the comparison with scAEGAN. The AE-Concat architecture consists of two encoders of one hidden layer, concatenated and projected down to the bottleneck layer. The first encoder takes the input from the first domain, and the second encoder takes input from the second domain. The first encoder and second encoder dimensions are 30 each, summing up to 60 dimensions after concatenating, projected down to a low dimensional space of 50 dimensions in the bottleneck layer. This layer contains the integrated low-dimensional representation of the two domains. ReLU is used as an activation function and a dropout value of (0.2). This concatenated network is trained with Adam optimizer with a learning rate of *lr* = 0.0005 for 200 epochs using a batch size of 16. The concatenated AE uses the mean square error as a loss function to minimize the input and output loss.

### Overview of the evaluation metrics

Firstly, the overlap between datasets before and after integration was visually assessed in low-dimensional representations using the UMAP R package v0.2.3.1. In the case of scAEGAN, integration quality was measured by transferring labels between domains. A support vector machine is first trained to classify cell types in one domain using the cluster assignments obtained from Louvain clustering as implemented in Seurat3. This step is followed by the prediction of cell type in the other domain and a comparison with the original clustering in this domain. In the case of AE integration, direct label transfer between input space and low-dimensional representation of the integrated dataset is not applicable. Accordingly, cell types are again assigned to input and integrated datasets via clustering with the Louvain algorithm and are then directly compared.

Furthermore, Seurat was used to transfer labels using its TransferData function. Cell type assignments, i.e., clusterings, are compared using the Adjusted Rand Index (ARI) in R package pdfCluster v1.0.3. and the Jaccard Index (JI) in R package clusteval v0. In addition to ARI and JI, we used Preserved Pairwise Jaccard Index (PPJI), a non-symmetric distance metric between two clusterings, for evaluating the clustering results.

Since Seurat is the most widely used tool, we compare our integration results with Seurat version 3 and 4 for the different library protocols on paired/unpaired data.

#### Jaccard Index (JI)

The JI calculates a 2 by 2 contingency table of agreements and disagreements between the two finite subsets and evaluates the stability of clustering. Given two subsets Ai and Bj, the JI is computed as:

$$JI\left(A_{i},B_{j}\right)=\;\frac{\left|\;A_{i}⋂B_{j}\right|}{\left|\;A_{i}\;⋃B_{j}\right|}$$

#### Adjusted Rand Index (ARI)

The ARI measures the similarity between the two partitions of the same datasets by the proportion of the agreement between the two partitions. The metric is adjusted for chance, such that the independent have an expected index of zero and identical partitions have an ARI equal to 1. The ARI is computed as:

$$ARI=\;\frac{\Sigma_{ij}\left(\frac{n_{ij}}{2}\right)-\left[\;\Sigma_{i}\left(\frac{a_{i}}{2}\right)\Sigma_{j}\left(\frac{b_{j}}{2}\right)\right]/\left(\frac{n}{2}\right)}{\frac{1}{2}\;\left[\;\;\Sigma_{i}\left(\frac{a_{i}}{2}\right)+\Sigma_{j}\left(\frac{b_{j}}{2}\right)]-[\;\Sigma_{i}\left(\frac{a_{i}}{2}\right)\Sigma_{j}\left(\frac{b_{j}}{2}\right)\right]/\left(\frac{n}{2}\right)}.$$

Where, *n*_*ij*_ refers to the number of common cells between two partitions and *a*_*i*_ = ∑_*k*_ (*n*_*ik*_), *b*_*j*_ = ∑_*k*_ (*n*_*jk*_) are the number of cells in estimated cluster i and in true cluster j, respectively.

*PredRNA*. RNA prediction was carried out by training the cGAN on the scRNAseq/scATACseq paired dataset and predicting on the held-out set.

Evaluation for the quality check was performed by computing Pearson correlation between each pair of cells from predicted RNA and original RNA training input data. This computation was performed using cor function from the stats package.

*Clustering for integrated and independent omic modalities*. The Seurat Louvain clustering implementation was used for all of the clustering analysis [26]. Various inputs are considered depending on the analysis:

Single-cell RNA-seq data: PCA components.

Single-cell ATAC-seq data: LSI components.

Cells were clustered based on shared components generated by the methods studied (scAEGAN, Seurat3, Seurat4).

For integrated subspaces, the Louvain resolution has been set to the default value of 0.6. The number of nearest neighbors has been used as K = 20.

### Data

For developing and testing this computational approach’s performance and quality, four different datasets (same/different modality, library preparation protocols) have been used. The summary of the datasets used is given in Table 1.

**Table 1:** Dataset summary providing data modality, sequencing platform, and number of cells employed for integration after pre-processing.

Data set	Platform	Data Modality	No of Cells
Pancreatic-Islet-Cells (Korsunsky et al., 2019)	Fluidigm C1	scRNA-seq	638
	CelSeq		946
	CelSeq2		2238
	SmartSeq		2355
Kowalczyk(Old/Young) (Johansen & Quon, 2019)	SmartSeq	scRNA-seq	524 / 498
SymSim (Zhang et al., 2019)		scRNA-seq	600
Chen(scRNA-seq/scATAC-seq) (Chen et al., 2019)	SNARE-seq	scRNA-seq/scATAC-seq	6735

#### Simulated datasets

Two datasets containing 600 cells from 5 populations and with 3000 genes each were simulated using SymSim [27] with the ‘Phyla5’ tree and the following parameters: nevf 35, evf_type ‘continuous’, n_de_evf 5, sigma 0.5, gene_effect_prob 0.5, gene_effect_sd 0.2, alpha_mean 0.05, alpha_sd 0.02, depth_mean 5e4, depth_sd 3e3. For one of these datasets, branch lengths of the ‘Phyla5’ tree was slightly modified.

Two more datasets were simulated for analysis to examine the sufficiency of scAEGAN when there is a cell type unbalance in two datasets. For dataset A, we simulated multiple versions with all cells, 100, 50, and 10 cells for the largest cluster, and for dataset B, we opted to remove the largest cluster, which had about 200 of the 600 genes in it.

#### Real datasets

The pre-processed mouse hematopoietic stem cell dataset of young and old individuals presented by was downloaded from https://github.com/quon-titative-biology/scalign [8]. Seurat’s NormalizeData and ScaleData functions were used to scale and center the count matrix after normalizing it to TP10K.

Four human pancreatic islet cell datasets sequenced using different platforms were obtained pre-processed as described in from https://github.com/immunogenomics/harmony2019 [9]. Raw read count matrices were scaled and normalized using Seurat v3 prior to integration.

*scRNAseq/scATACseq paired dataset*. We selected an existing paired scRNA-scATAC dataset from the SNARE-seq protocol (a droplet-based single nucleus over mRNA expression and chromatin accessibility sequencing) [28]. The data was downloaded from GSE126074. The preprocessing applied to this dataset is as follows:

Quality filter—low-quality features: removes low-quality features and cells from both modalities. We excluded all cells with an overall abundance level of "number of features per cell" and "number of counts per cell" less than quantile 0.1 and greater than quantile 0.9. For mRNA (ATAC), minimum abundance filtering was used: genes (peaks) profiled in less than 4 cells (3 cells) and cells with fewer than 201 genes quantified were filtered. There was no requirement for a certain number of peaks per cell. Following quality and abundance filtering, we considered a total of 8,086 cells for scRNA and 8,214 cells for scATAC adult samples for analysis.

*ATAC-derived gene activity*. To compute ATAC-derived gene activity, the Seurat3 ’CreateGeneActivityMatrix’ function with "upstream = 2000" bases was used. In addition, the GRCh38 genome was used as a reference to later identify marker genes across the integrated expression subspace.

Quality filter—mitochondrial: 5 percent mitochondrial filtering was used for the expression matrices of scRNA and scATAC, with activity from peaks used in the ATAC case.

*Component parameters*. For scRNA reduction, 15 principal components (PCA) were chosen, and 50 latent semantic indexing components (LSI) were chosen for scATAC.

The final number of cells: from the resulting pipeline, a total of 6,735 paired cell profiles were considered for the downstream analysis.

*Integration*. On this dataset, Seurat 3 (unpaired) and Seurat 4 (paired) were used to generate a reference integrated version for further processing and later integration. Using standard normalization and integration guides for Seurat3 and Seurat4 Weighted Nearest Neighbor Analysis vignettes (Hao et al., 2021). In Seurat 3, the FindTransferAnchors function was used to generate anchorsets using RNA as the reference and ATAC as the query modalities, with CCA as the reduction method. This was followed by the TransferData function, where the anchorset generated was used to transfer the RNA derived information into the ATAC modality using LSI dimensional reduction for the weighting anchors. Seurat 4 was used to identify multi-modal neighbors using the FindMultimodalNeighbors function.

## Results

### A novel architectural design for single-cell multi-data set analysis

We propose an integrated AE and cGAN architecture (Fig 1a), allowing the integration of scRNAseq data from different datasets. A particular experiment in a given data domain produces a cell count matrix, which is then fed into the encoder of the AE to condense it into a lower-dimensional latent representation. The objective of the decoder is to reconstruct the input from the latent representation. This defines a reconstruction loss function for the AE (for details and hyperparameters, see Material and methods). This procedure results in two datasets from the same system of interest, each with a lower-dimensional latent representation. The cGAN’s task is to learn a non-linear mapping between latent space representations using a cycle consistency loss (Material and methods). This procedure constitutes a robust, flexible, and unifying neural network architecture supporting several integration scenarios, such as between scRNA-seq datasets from replicates, library protocols, and data modalities.

### scAEGAN preserves the cell identity and accurately identifies the cell clusters

To evaluate this concept’s viability and performance, we first tested the scAEGAN by simulating scRNA-seq data using SymSim [27]. Cells were generated according to a cell population tree, defining several clusters with different distances. This procedure generated two datasets. Each dataset in this simulation had five continuous clusters. In a continuous mode, the cells are positioned along the edges of the tree with a small step size (which is determined by branch lengths and the number of cells. Each dataset has 600 cells and 3000 genes simulated with 20 External Variability Factors (EVFs), 12 differential EVFs, and a sigma of 0.4 (Material and methods, S1 Fig). The number of clusters is preserved in the AE-derived low-dimensional embedding. Visual comparison with the translated version of the other domain reveals good agreement (Fig 1b). We quantified the integration quality by measuring the transfer of labels between the data domains. To this end, we used an SVM to classify cell types in one domain using cluster assignments. Next, we measured the transferred cell identity agreement with the original identity using the Jaccard Index (JI) and Adjusted Rand Index (ARI) (Material and methods). The JI calculates 2 by 2 contingency table of agreements and disagreements of the corresponding two vectors of comemberships. Comembership is defined as the pairs of observations that are clustered together. In contrast, ARI measures the similarity between the two alternate partitions of the same datasets by the proportion of agreements between the two partitions. The higher the ARI value, the more accurate the clustering, and when the cluster is perfectly matched to the reference criteria, the ARI score equals 1. The scAEGAN preserved the transferred cell identity agreement with the original identity (Fig 1c and 1d).

### scAEGAN integrates datasets across different library protocols

**Fig 2:**
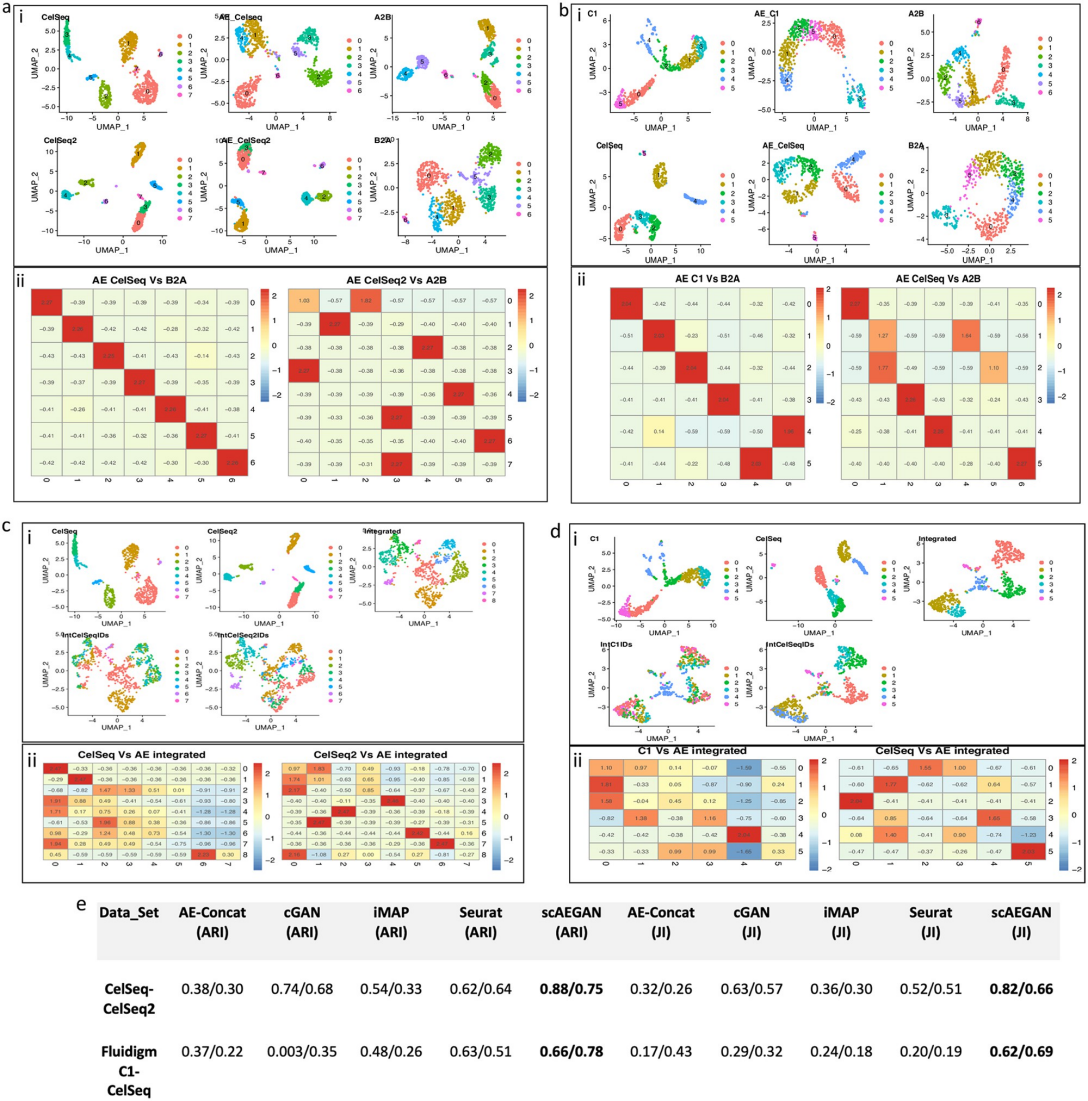
Integration results of scAEGAN with across platforms data (CelSeq, CelSeq2, Fluidigm C1) **a, b)** scAEGAN results show better translation of the domains, while maintaining the cluster granularity in the respective domains, while integrating the datasets from CelSeq, CelSeq2 and Fluidigm C1. Integration results of AE-Concatenated with across platforms data (CelSeq, CelSeq2, Fluidigm C1) and its quantitative comparison with scAEGAN, **c, d)** The results from the AE-Concatenated shows its bad performance while integrating the datasets from CelSeq, CelSeq2 and Fluidigm C1, **e)** scAEGAN results shows its outperformance as compared to AE-Concatenated, iMAP, Seurat and cGAN for integrating data across different platforms.

We systematically assessed the ability of scAEGAN-derived feature representations to integrate different library protocol datasets. To this end, we evaluate and compare scAEGAN with Seurat3 as Seurat3 has demonstrated that it can integrate two datasets using different library protocols. It has performed better than Liger [11] and scMerge [13] when integrating datasets across different single-cell RNA sequencing protocols [29]. We evaluated and compared scAEGAN with Seurat 3 using an easier translation task using ARI and JI as evaluation metrics. We first analyzed the case where we have two versions of the same protocol (CelSeq to CelSeq2) and contrasted this with the more challenging task of integrating two different protocols, e.g., fluidigm F1 with CelSeq. Seurat3 performed well on the easy task (0.62 ARI, 0.52 JI, Fig 2e). Yet, scAEGAN outperformed Seurat 3 in this task (0.88 ARI, 0.82 JI, Fig 2a, 2b and 2e). Interestingly, the concatenated architecture (0.38 ARI, 0.32 JI, Fig 2c–2e) was outperformed by Seurat3. Notably, even the cGAN outperformed Seurat3 (Fig 2e). For the more challenging task (fluidigm F1 with CelSeq), while the performance of scAEGAN dropped (0.66 ARI, 0.62 JI), it still outperformed all other methods and architectures. Even with this challenging task, scAEGAN obtained finer granularity in terms of added value to clustering (Fig 2b). We noted that the concatenated and cGAN outperformed Seurat3 in this task. Similar results were obtained in integrating Celseq2 and SMARTseq (S3 Fig). scAEGAN outperformed (0.78 ARI, 0.69 JI) all other methods and architectures. We also evaluated the scAEGAN’s robustness by reducing the no of cells by randomly selecting a % of cells (20, 40, 60, and 80) and computing the ARI for each case. We observed that reducing the number of cells diminishes the performance of Seurat3 and AE-concatenated. Interestingly, when reducing the number of cells, scAEGAN outperforms Seurat3 and AE-concatenated (S3 Fig) significantly, thus suggesting the better robustness of scAEGAN compared to Seurat3 and AE-concatenated.

**Fig 3:**
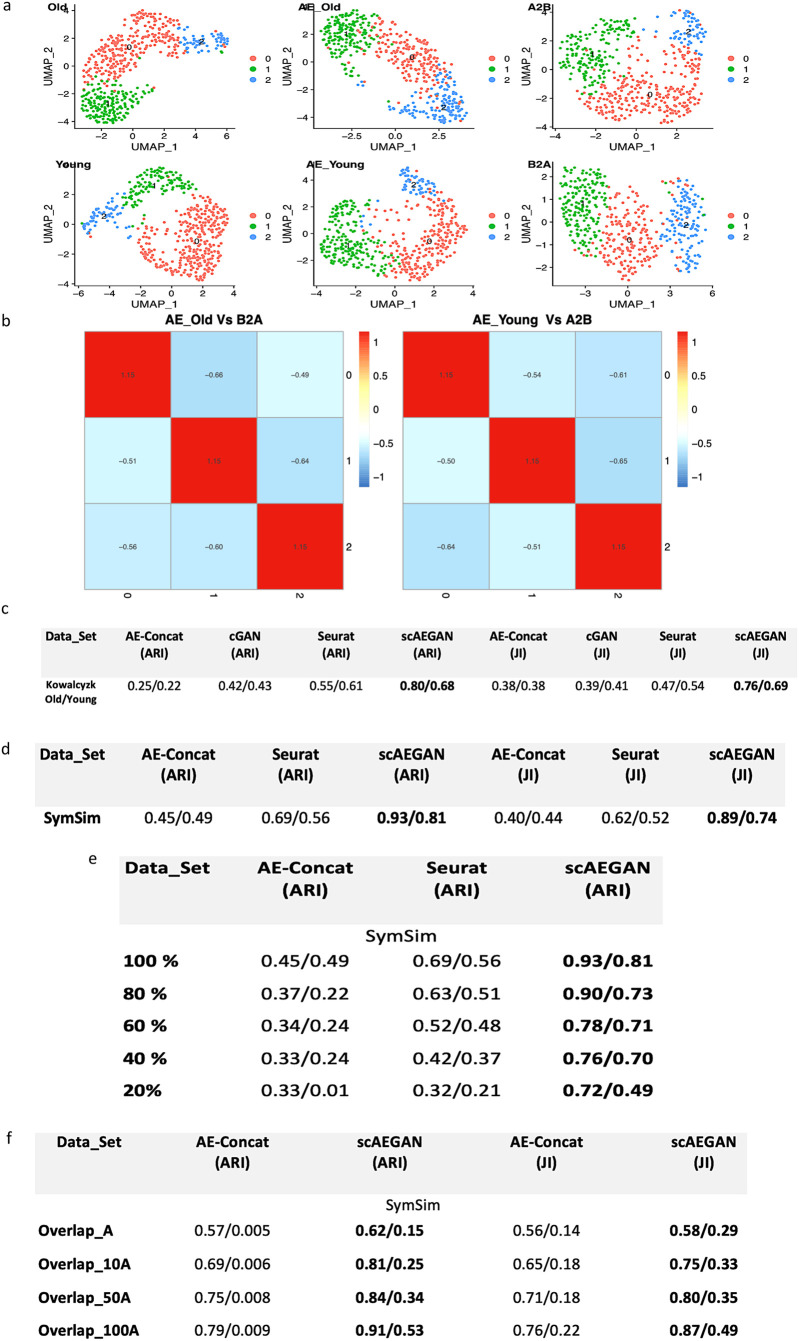
scAEGAN shows robust performance, while integrating datasets from the same platforms, **a)** scaAEGAN outperforms other methods for integrating a real scRNA-seq SMARTseq dataset from two mouse strains (Old and Young). A2B and B2A are the integration results of the Old and Young mice datasets with the latent spaces of input dataset from these two mouse strains, **b)** shows the heatmap of the cluster similarity of latent space of old mice dataset (AE_Old) with the translated domain B2A and likewise for young mice dataset (AE_Young Vs A2B) and **c)** shows the ARI and JI values for Old and Young mice dataset, depicting the outperformance of scAEGAN as compared to other methods. **d)** ARI and JI values for both simulated datasets A and B, depicting the outperformance of scAEGAN with AE-concatenated and Seurat, **e)** scAEGAN performs better, even the certain percentage of cells are removed from two datasets, **f)** scAEGAN shows robust performance, when there is an imbalance of cell types in two datasets (denoted by No overlap with A (cluster present in dataset A only and not in dataset B), 10_A(10% of cells removed in dataset A from cluster 6_1) and likewise for 50_A and 100_A respectively.

To assess the GAN cycle consistency loss contribution, we fused the two latent representations by concatenation instead of learning a mapping (Material and methods). This caused a dramatic drop in performance (0.93 to 0.45 ARI, 0.89 to 0.40 JI, Fig 3d). Notably, this significant drop occurred despite the simplified situation of well-separated simulated clusters where the latent space’s dimensionality was the same for the two data domains. Furthermore, the analysis using simulated data was repeated for several cases; all results followed the above observations (Material and methods, S1 Fig). Therefore, we concluded that the proposed architecture is sufficient to perform the integration. Furthermore, the analysis also demonstrated the importance of learning a non-linear relationship between the two latent spaces.

Next, we asked whether we could learn to integrate the input datasets using a cycleGAN without employing an Autoencoder first to project the data into a latent space. This would conceptually correspond to a pixel-by-pixel translation between images. The cycleGAN performed better on the simulated datasets (0.99 ARI, 0.92 JI). But when using a real scRNA-seq SMARTseq dataset from two mouse strains [8], a reduced performance compared to scAEGAN (0.80 to 0.42 ARI, 0.76 to 0.39 JI, Fig 3a–3c). Both values represent the corresponding ARI and JI values for dataset A and B, respectively.

Interestingly, the dataset contains several less-informative PCA components likely representing noise in the original data, making it challenging to learn a stable non-linear mapping between the two domains (S2 Fig). The effect of AE training on the two mouse strain datasets retains the most informative PCA components. It removes the components with noise, thus facilitating a linear stable mapping between the two domains (S2 Fig). We also evaluated the robustness of scAEGAN in a simulated setting when we had an imbalance of cell types in two datasets. The imbalance setting ranges from having dataset B with no cluster 6_1 i.e., (cluster 6_1 present in dataset A but not in dataset B), to removing 10, 50, and 100% cells from that cluster from dataset A. (100_A) represents that 100% of cells are removed from cluster 6_1 from dataset A, thus depicting that both dataset A and dataset B doesn’t have this cluster 6_1. (50_A) represents, 50% of cells from cluster 6_1 is removed from dataset A and likewise (10_A) represents that 10% of cells from cluster 6_1 is removed from dataset A respectively. (No overlap with A) represents that, the there is no cluster 6_1 in dataset B, while this cluster is in dataset A. Here scAEGAN performed well (0.62 ARI, 0.58 JI) compared to other methods and architectures (Fig 3f). To further evaluate the robustness of the scAEGAN, we reduced the number of cells by randomly selecting a fixed percentage of cells (20,40,60 and 80%) in the simulated dataset. Here scAEGAN outperforms Seurat3 (Fig 3e), thus suggesting the robustness of scAEGAN compared to Seurat3. Finally, we compared our analysis of the simulated data and the mouse dataset with Seurat3. Overall, the scAEGAN was more successful than Seurat 3 in transferring the labels correctly, whereas Seurat3 was better than the concatenated architecture, thus further supporting the importance of cycleGAN learning.

### scAEGAN outperforms existing methods for the integration of paired and unpaired multi-omic datasets

**Fig 4:**
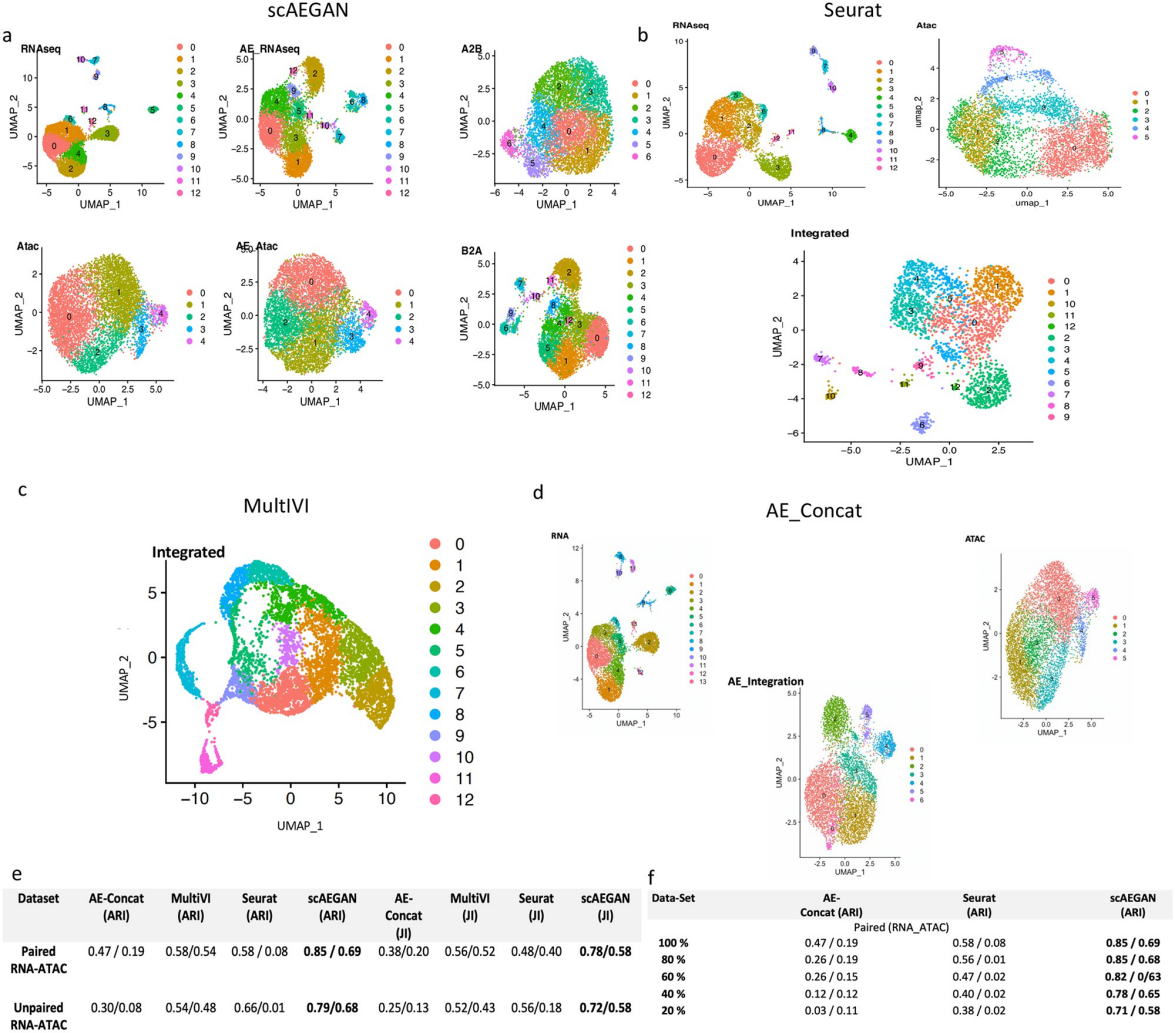
Multi-modal integration results of scAEGAN with paired scRNA-seq and scATAC-seq data. The unpaired case is simulated by randomizing the pairing information, **a, b)** 2D UMAP visualization of integration results from scAEGAN and Seurat with the input domains (Rna, Atac) respectively, depicting that scAEGAN preserving the cluster identity in the translated domains. **c, d)** 2D UMAP visualization of integration results from MultiVI and AE_Concat with the input domains (Rna, Atac) **e)** scAEGAN outperforms AE-Concatenated, MultiVI and Seurat 4, even when discarding the pairing information between the two modalities **f)** scAEGAN shows robust performance, even when certain % of cells are removed from each modality.

Aiming for generality, we investigated the integration of multi-omic datasets. To this end, we integrated scRNA-seq and scATAC-seq data as a case study. When the scRNA-seq and scATAC-seq data are collected from different cells, referred to as unpaired data, it also includes the challenge of having different samples. Both data modalities are collected from the same cell in the paired case. Thus, the integration of scRNA-seq with scATAC-seq data could be either paired or unpaired. Recent progress has mainly targeted unpaired data. Tools such as Seurat3 and MOFA+ have demonstrated promising results. A recent upgrade, Seurat4, is the first attempt to our knowledge targeting the paired data-integration challenge. We evaluated the architectures using paired (Fig 4a–4d) and unpaired data. As for the previous settings we used the Jaccard Index and Adjusted Rand Index as quality measures for quantifying the integration quality. Interestingly, scAEGAN outperforms Seurat 3, Seurat 4, and MultiVI, even when discarding the pairing information between the two modalities (Fig 4e). To further assess the robustness of scAEGAN, we evaluated the performance of scAEGAN by removing the % of cells in paired data. We observed that the performance of Seurat4 decreases with the number of cells compared to the scAEGAN. On the other hand, the scAEGAN outperforms Seurat4 (Fig 4f), thus suggesting better robustness than the Seurat4.

### scAEGAN facilitates predicting one modality from another modality

To further investigate the efficacy of scAEGAN, we attempted to predict one modality from another modality. We trained the scAEGAN on scRNA-seq and tried to predict the scATAC-seq. To this end we divided our scRNA-seq and scATAC-seq data into training(*n* = 53850) and testing (n = 1350) sets and trained scAEGAN jointly on scRNA-seq and scATAC-seq training sets. After training predictions were inferred from the test set. We used Pearson correlation as an evaluation metric as used in Babel, for cross-domain inference between the empirical expression(scRNA-seq) and the scAEGAN’s inferred (translated expression) between each pair of cells. scAEGAN outperforms Babel, where scAEGAN achieved a Pearson correlation (0.60) compared to Babel (0.55).

## Discussion

Recent technological advances in single-cell genomics (SCG) have set the stage to discover, catalog, and characterize cell types at an unprecedented level using various profiling techniques and library protocols. In addition, such community efforts have increasingly produced single-cell atlases at an unprecedented resolution and scope [30]. Yet, we need to synthesize data from various sources to achieve a more holistic understanding of cellular identity, diversity, and function. However, integrating data from different data modalities, samples, and library protocols when studying a specific question or biological system is an unprecedented challenge [31]. Several highly specialized machine learning techniques address, as a rule, a narrow challenge, such as how to integrate different samples of scRNA data. Yet, when studying a specific question or biological system, there is a need to integrate data originating from one or more data modalities, different library protocols, and paired or unpaired data.

Moreover, the investigator wants to predict missing data or data modalities from the available data samples. Such predictions are helpful since they can be subject to validation in downstream experiments. However, it is challenging and time-consuming to navigate and potentially combine different tools and their results to perform a holistic integrative and predictive biological analysis.

To address this challenge, we developed scAEGAN, a unifying end-to-end unsupervised single-cell data integration and predictive method combining an AE architecture for efficient representation of scRNA-seq data with a CycleGAN network for translation across datasets. We demonstrate the sufficiency in that such a unifying machine learning architecture can achieve state-of-the-art or better performance by tackling seemingly “different” integration challenges. Anchoring-based methods, such as Seurat [3], have a strong domain of applicability and performance when the different datasets are “close” or “similar”. This result is natural since the method is predicated on the assumption of “shared” anchors. Yet, the anchoring approach is limited when the datasets are too dissimilar or when there is a need to perform predictions out of the sample. For example, as for the challenge of predicting scATAC data from scRNA, machine learning techniques such as Babel [7] are superior to the anchoring approach. Yet, thus far, machine learning methods such as Babel have not yet been able to reach the performance of Seurat on a task such as clustering and integrating unpaired omics data. Here we find that scAEGAN is much more robust against sparsity in data than the anchoring technique when different datasets are similar. Notably, scAEGAN surpasses the current state-of-the-art technique for predicting out-of-the-sample data modalities. Our evaluations using the concatenated AE support the interpretation that the critical reason for our success is that the AE respects each sample’s uniqueness and protocol. The outcome of this evaluation makes sense since such a procedure preserves the biological signal instead of diluting the original signal by forcing differences in datasets to be reduced. In contrast, our novel architecture allows the cGAN network to exploit the similarity in the data distributions in the latent space. Thus, since we do not require similarity in the original dataspace, we can learn to map the latent space across different conditions, thus enabling a predictive capacity. An interesting challenge for future work is to further generalize our approach such that it can handle say N number of different modalities, paired or unpaired. In the current formulation, we would need to learn the mappings between the latent spaces corresponding to the different modalities. That would most likely require either an extension of the cycleGAN learning or a more generalized architecture suitable for the task.

As the community progresses with developing powerful data integration methods, we may be able to revisit the early vision of system biology [32]. Combining rich multi-modal high-resolution single-cell data with data-driven integration techniques may enable mechanistic predictive modeling of cells and their interactions [33]. Whole-cell modeling has been challenging in the past. Still, being an attractive target in the system biology community. Part of the challenge is the model size and a large number of parameters [34, 35]. This could, in part, be mitigated by efficient integrative multi-modal models capturing the essence of the signal in the data. This would reduce the model size and the number of parameters. The attraction is that by using modeling based on integrated single-cell data, we can, on the one hand, reach fundamental insight into biological processes and begin to disentangle mechanisms of diseases [36]. Thus, it remains vital to explore how to integrate single-cell data into a coherent interpretable representation of cells and their interactions. We view the scAEGAN as one step towards this larger aim.

## Supporting information

S1 Fig Two datasets containing 600 cells from 5 populations and with 3000 genes simulated using SymSim (X. Zhang et al., 2019) with the ‘Phyla5’ tree and the following parameters: nevf 35, evf_type ‘continuous’, n_de_evf 5, sigma 0.5, gene_effect_prob 0.5, gene_effect_sd 0.2, alpha_mean 0.05, alpha_sd 0.02, depth_mean 5e4, depth_sd 3e3. (TIFF)

S2 Fig Jackstraw plot showing the informative principal components from the young and old mice as well as simulated datasets. (TIFF)

S3 Fig Integration results with across platforms data from CelSeq2, SmartSeq and its quantitative comparison, **a)** scAEGAN results shows its outperformance as compared to AE-Concatenated, integrating data CelSeq2, SmartSeq platforms, **b)** The results from the AE-Concatenated shows its bad performance while integrating the datasets from CelSeq2, SmartSeq platforms, **c)** scAEGAN results shows its outperformance as compared to AE-Concatenated, Seurat and cGAN for integrating data across different platforms.
(ZIP)
