# Supplementary figures and images for: scAEGAN: Unification of single-cell genomics data by adversarial learning of latent space correspondences
Source: PLoS One. 2023 Feb 3;18(2):e0281315. doi: 10.1371/journal.pone.0281315 (PMC9897517; doi:10.1371/journal.pone.0281315)

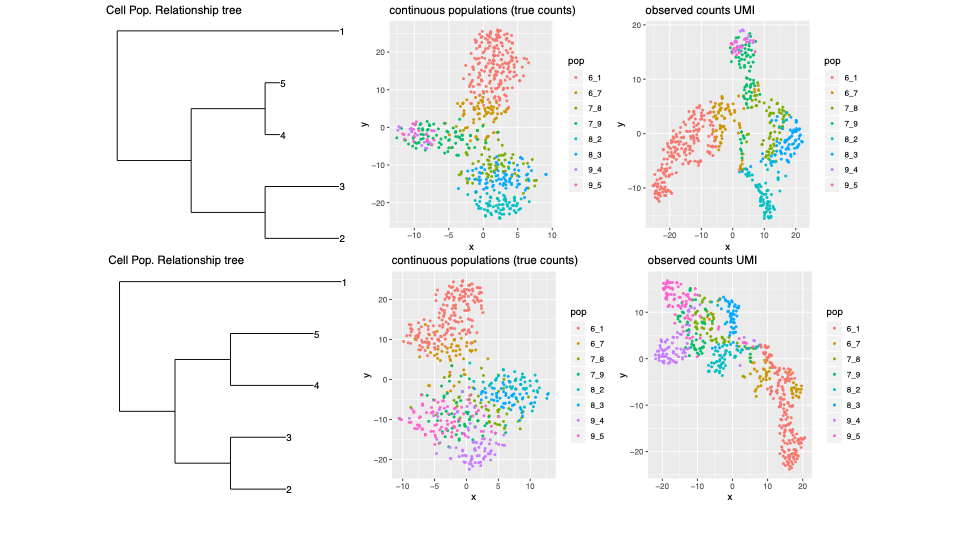

Supplement: S1 Fig — (TIFF) [file pone.0281315.s001.tiff]

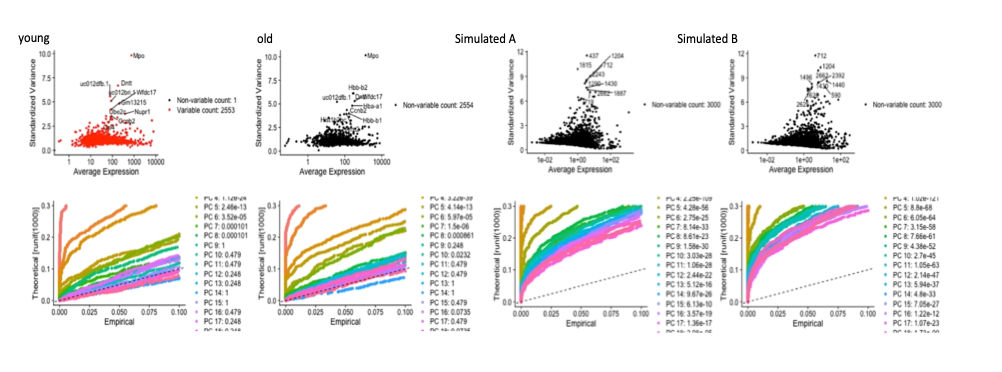

Supplement: S2 Fig — (TIFF) [file pone.0281315.s002.tiff]

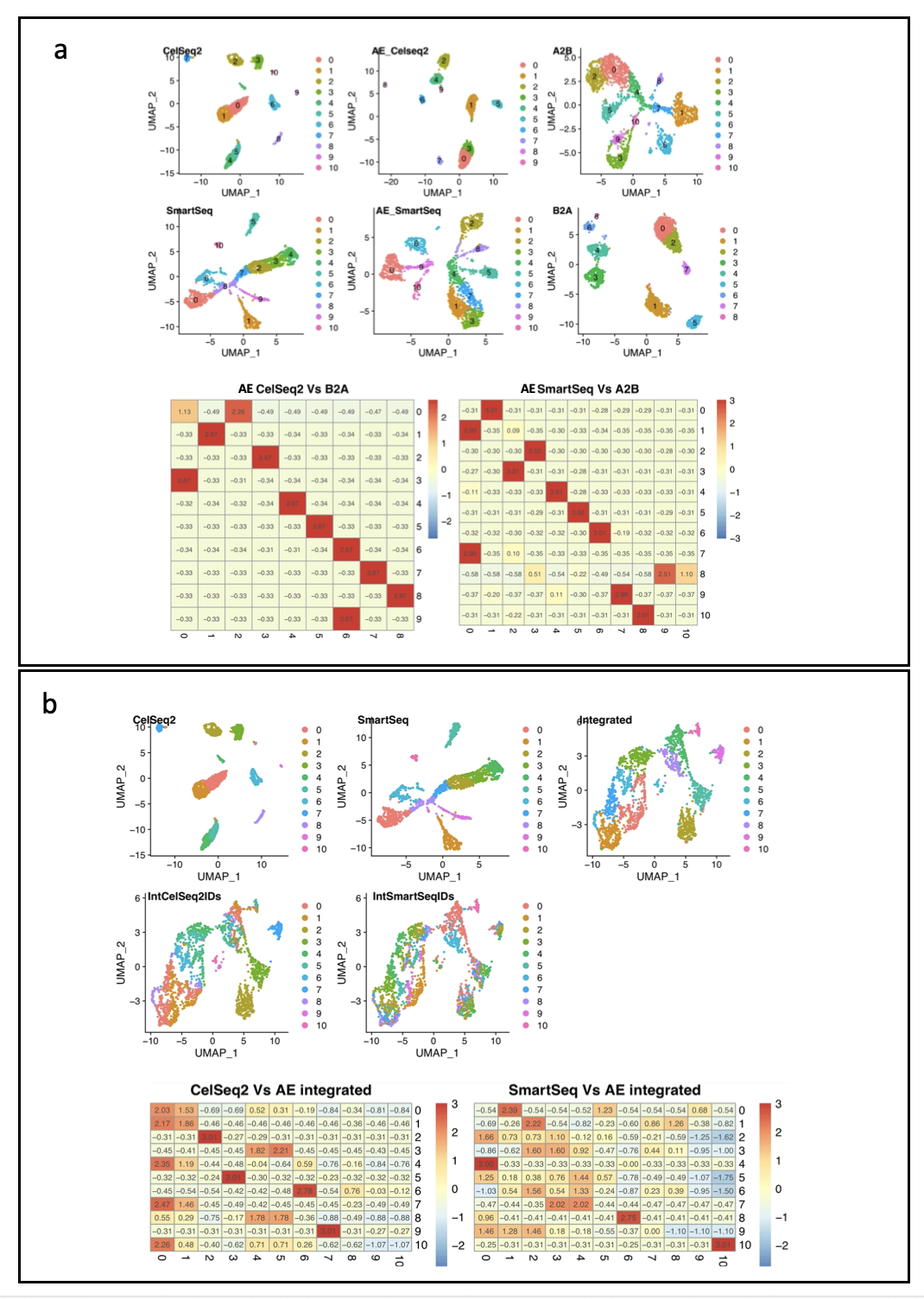

Supplement: S3 Fig — Integration results with across platforms data from CelSeq2, SmartSeq and its quantitative comparison, a) scAEGAN results shows its outperformance as compared to AE-Concatenated, integrating data CelSeq2, SmartSeq platforms, b) The results from the AE-Concatenated shows its bad performance while integrating the datasets from CelSeq2, SmartSeq platforms, c) scAEGAN results shows its outperformance as compared to AE-Concatenated, Seurat and cGAN for integrating data across different platforms. (ZIP) [file pone.0281315.s003.zip › S3 Fig ab.tiff]
